# Signal regulatory protein alpha blockade potentiates tumoricidal effects of macrophages on gastroenterological neoplastic cells in syngeneic immunocompetent mice

**DOI:** 10.1002/ags3.12205

**Published:** 2018-09-10

**Authors:** Tomoyuki Abe, Yuka Tanaka, Jinlian Piao, Naoki Tanimine, Naohide Oue, Takao Hinoi, Noel Verjan Garcia, Masayuki Miyasaka, Takashi Matozaki, Wataru Yasui, Hideki Ohdan

**Affiliations:** ^1^ Department of Gastroenterological and Transplant Surgery Graduate School of Biomedical and Health Sciences Hiroshima University Hiroshima Japan; ^2^ Department of Molecular Pathology Hiroshima University Graduate School of Biomedical Sciences Hiroshima Japan; ^3^ Division of Molecular Oncology Department of Surgery Institute for Clinical Research National Hospital Organization Kure Medical Center & Chugoku Cancer Center Hiroshima Japan; ^4^ Faculty of Veterinary Medicine, Immunobiology and Pathogenesis Research Group Altos de Santa Helena University of Tolima Ibague Colombia; ^5^ Institute of Academic Initiatives Osaka University Suita Japan; ^6^ MediCity Research Laboratory University of Turku Turku Finland; ^7^ Division of Molecular and Cellular Signaling Department of Biochemistry and Molecular Biology Kobe University Graduate School of Medicine Kobe Japan

**Keywords:** CD47, phagocytosis, SIRPα, syngeneic mouse model

## Abstract

**Aim:**

Immunotherapies blocking the CD47‐SIRPα pathway by targeting CD47 enhance macrophage phagocytosis of neoplastic cells in mouse models. As SIRPα exhibits relatively restricted tissue expression, SIRPα antagonists may be better tolerated than agents targeting CD47, which is ubiquitously expressed in many tissues. Here, we investigated the therapeutic impact of monoclonal antibodies (mAbs) against CD47 and/or SIRPα on gastroenterological tumors in syngeneic immunocompetent mouse models.

**Methods:**

We used in vitro and in vivo phagocytosis assays in C57BL/6J (B6) mice to investigate anti‐CD47/SIRPα mAb effects on Hepa1‐6 and CMT93 originating from B6 mice. The influence of these mAbs on macrophage transmigration was also assessed. To investigate anti‐SIRPα mAb therapy‐induced inhibitory effects on sporadic colon cancer growth, we used a *CDX2P9.5‐NLS Cre;APC* ^*+*^ */FLOX* (*CPC‐APC*) mouse model.

**Results:**

Systemic anti‐SIRPα mAb administration significantly increased Hepa1‐6 and CMT93 cell susceptibility to macrophage phagocytosis, both in vitro and in vivo. Conversely, similarly administered anti‐CD47 mAb did not promote macrophage phagocytosis of target cells, whereas cells incubated with anti‐CD47 mAb prior to inoculation were more susceptible to macrophage phagocytosis. In vitro cell migration assays revealed that binding with anti‐CD47 mAb inhibited macrophage transmigration. Anti‐SIRPα mAb treatment inhibited tumor progression in *CPC‐APC* mice and significantly improved overall survival. Anti‐CD47 mAb administration in vivo eliminated the phagocytosis‐promoting CD47 blockade effect, probably by inhibiting macrophage transmigration/chemotaxis. In contrast, anti‐SIRPα mAb exhibited enhanced macrophage phagocytic activity and marked anti‐tumor effects against gastroenterological malignancies.

**Conclusion:**

SIRPα mAb augmentation of macrophage phagocytic activity may represent an effective treatment strategy for human gastrointestinal tumors.

## 1 INTRODUCTION

The integrin‐associated protein CD47 is a ubiquitously expressed cell surface glycoprotein that serves as a ligand for signal regulatory protein (SIRP) α (also known as CD172a, SHPS‐1), an immune inhibitory receptor on macrophages. CD47 and SIRPα constitute a cell–cell communication system (the CD47‐SIRPα system) that is implicated in negative regulation of phagocytosis by macrophages. The intracellular SIRPα region possesses typical immunoreceptor tyrosine‐based inhibitory motifs that are phosphorylated after ligation, resulting in cytosolic protein tyrosine phosphatase SHP‐1 and/or SHP‐2 recruitment and activation.^1^, ^2^, ^3^, ^4^ For certain cells (i.e., erythrocytes, platelets, or leukocytes), surface CD47 can protect against macrophage‐mediated phagocytosis by binding to the inhibitory macrophage receptor SIRPα.^5^, ^6^, ^7^ The interaction of SIRPα on macrophages with CD47 on leukemia and even solid tumor cells also prevents phagocytosis of such neoplastic cells. Immunotherapies intended to block the CD47‐SIRPα pathway have been proven to augment macrophage phagocytosis of several different types of neoplastic cells, primarily in mouse xenogeneic and even syngeneic transplantation models^8^, ^9^, ^10^, ^11^, ^12^, ^13^; however, their preclinical activities in further clinically relevant models remain to be elucidated.

We have previously shown that the interspecies incompatibility of CD47 is responsible for human macrophage‐mediated phagocytosis of xenogeneic porcine cells. Porcine CD47 does not induce SIRPα tyrosine phosphorylation in human macrophages, and porcine cell manipulation for expression of human CD47 markedly reduces their susceptibility to human macrophage‐mediated phagocytosis.^14^ Beyond such interspecies incompatibility, the non‐obese diabetic (NOD) mouse strain provides a better background for human leukocyte and cancer cell engraftment than those of other strains with equivalent immunodeficiency‐related mutations owing to the stronger engagement of the SIRPα inhibitory receptor with human CD47, preventing engulfment of human grafts.^15^ By using NOD mice with multiple immunodeficient phenotypes, anti‐human CD47 monoclonal antibodies (mAbs), as inhibitors of the CD47‐SIRPα interaction, have been shown to display anti‐tumor activity against various human cancers including leukemia and solid tumors.^9^, ^13^ In these xenograft models, anti‐human CD47 mAb targets human CD47 on inoculated human cancer cells but does not recognize mouse CD47 ubiquitously expressed on the host mouse cells. This has raised concerns that effects caused by CD47 binding to multiple other ligands, such as integrins and thrombospondin, which govern a number of processes in normal tissues, might be overlooked. Although examined in mouse models with locally and mainly subcutaneously transplanted syngeneic tumors,^8^, ^9^, ^10^, ^11^, ^12^, ^13^ this concern remains to be investigated following systemic tumor inoculation. In the present study, we investigated the anti‐tumor effects of mAbs targeting CD47 or SIRPα, which exhibits relatively restricted tissue expression, by use of a mouse model in which syngeneic gastrointestinal cancer cells are intraperitoneally inoculated and a gene‐targeting mouse model in which colon cancers grow sporadically.

## 2 MATERIALS AND METHODS

### 2.1 Mice

C57BL/6J (B6) mice were purchased from Clea Japan (Osaka, Japan). *CDX2P9.5‐NLS Cre* and *APC* ^*LOX/FLOX/FLOX*^ mouse embryos were obtained from the University of Michigan. These embryos were transferred to pseudopregnant B6 mice, and those carrying the *CDX2P9.5‐NLS Cre* recombinase transgene and a *loxP*‐treated *APC* allele (*CPC‐APC* mice) primarily developed colorectal adenocarcinomas from 9 weeks.^16^

### 2.2 Cell cultures

The Hepa1‐6 B6 murine hepatoma cell line, CMT93 B6 colon cancer cell line and Huh7 human hepatoma cell line were purchased from the American Type Culture Collection (Manassas, VA, USA). Cells were cultured in Dulbecco's modified Eagle's medium (Gibco, NY, USA) containing 10% fetal bovine serum with 5 μmol/L 2‐mercaptoethanol (Katayama, Osaka, Japan), 10% HEPES buffer solution (Gibco), and 100 units/mL penicillin and 100 μg/mL streptomycin (Gibco) at 37° Ϲ under a humidified atmosphere of 5% CO_2_ in air.

### 2.3 Anti‐SIRPα and anti‐CD47 mAbs

Anti‐SIRPα mAb was prepared from My‐1 hybridoma cells as previously reported.^17^ Hybridoma cells were grown in hypoxanthine‐aminopterin‐thymidine medium supplemented with IL‐6, and culture supernatants were screened for Abs reactive to SIRPα‐expressing leukocytes by flow cytometry (FCM). Anti‐SIRPα mAb was prepared in ascitic fluid from ICR nu/nu mice, determined to be of the IgG type, and purified using protein G‐affinity chromatography.^17^ Miap301 hybridoma cells producing anti‐CD47 mAb were kindly donated by P. A. Oldenborg (Umeå University, Umeå, Sweden).^7^ Anti‐CD47 mAb was produced and analyzed in a similar manner as anti‐SIRPα mAb.

### 2.4 Lentiviral‐encoded small hairpin RNA (shRNA) knockdown of Hepa1‐6 cells

ShRNA constructs targeting knockdown of mouse CD47 or a GFP control were transduced into Hepa1‐6 and CMT93 cells as follows. Cells were seeded into 48‐well plates (BD Falcon, San Diego, CA, USA) and incubated at 37° C for 18–20 h in a humidified atmosphere with 5% CO_2_. Hexadimethrine bromide (Sigma‐Aldrich, St. Louis, MO, USA) was then added to each well. An appropriate amount of lentiviral particles at a suitable multiplicity of infection was also added to appropriate wells. Cells were incubated with the viral particle mixture at 37° C overnight. CD47 protein level knockdown was determined by staining with anti‐CD47 mAb (clone miap301) with fold knockdown calculated by determining the reduction of mean fluorescence intensity normalized over isotype‐control antibody. The following oligonucleotides were used to knockdown CD47 expression: shCD47#1 (CCG GCC CGT TCT GCT ACT TTG ATT TCT CGA GAA ATC AAA GTA GCA GAA CGG GTT TTT G) and shCD47#2 (CCG GCC CGT TCT GCT ACT TTG ATT TCT CGA GAA ATC AAA GTA GCA GAA CGG GTT TTT G).

### 2.5 In vivo mAb treatment in *CPC‐APC* mice

*CPC‐APC* mice originated from Apc^F/wt^ mice harboring a *CDX2‐Cre* transgene in which colorectal tumorigenesis is driven by *APC* allelic loss. These mice were administered intraperitoneal injections of either 400 μg/mouse of rat anti‐mouse IgG control Ab (Jackson ImmunoResearch, West Grove, PA, USA) or anti‐SIRPα mAb (My‐1) once weekly from the eighth week until the day of harvest. For tumor size evaluation, mouse colonoscopy was performed using a grading system according to tumor circumference: grade 1 (very small but detectable tumors) and grades 2–5 (tumors occupying up to one‐eighth‐ [grade 2]; a quarter‐ [grade 3]; half‐ [grade 4]; or more than half [grade 5] of the colonic circumference). We previously reported that the colonoscopy evaluation procedure tumor detection specificity was 1.00 and the sensitivity was 0.98.^18^ All mice were killed at week 20 to assess colorectal cancer development by histological analysis. To evaluate anti‐tumor effects, mice were euthanized at week 20 to assess colorectal cancer development via histological analysis. This experiment was independent from the survival experiment. The experimental schema is shown in supporting information (supplemental material and methods).

### 2.6 Flow cytometry

Cell suspensions were pre‐incubated with anti‐CD16/32 (2.4G2) mAb to block Fcγ II/III receptors and stained for 15 min with the following fluorochrome‐conjugated mAbs in a six‐color staining combination. For cell surface SIRPα expression analysis, we used PE‐labeled SIRPα mAb (P84, BD Pharmingen, San Diego, CA, USA). To analyze cell surface CD47 expression, we used purified anti‐CD47 mAb (miap301, BD Pharmingen), followed by a secondary anti‐rat polyclonal IgG‐biotin (BD Pharmingen) and then APC‐conjugated streptavidin. The following anti‐mouse fluorochrome‐conjugated mAbs were used (BD Pharmingen): PE‐labeled CD11b (M1/70), APC‐labeled anti F4/80 (BM3), and APC‐Cy7‐labeled anti‐CD19 (1D3). Dead cells were excluded from the analysis by light‐scatter and 7‐ADD staining. Cells were analyzed on a FACS Canto II cytometer (BD Biosciences, San Jose, CA, USA). Data were analyzed using FlowJo 7.6.5 (Tree Star Inc., Ashland, OR, USA). For human in vitro assay, APC‐labeled anti‐CD14 (61D3) was used.

### 2.7 In vitro phagocytosis assay

Target cells were labeled with 5 μmol/L carboxyfluorescein succinimidyl ester (CFSE) (Molecular Probes, Eugene, OR, USA), gently mixed, and incubated for 15 minutes at 37° C in a CO_2_ incubator protected from light.^19^ To prepare peritoneal macrophages, peritoneal cells were harvested from B6 mice after intraperitoneal phosphate buffered saline (PBS) injection, plated in a 24‐well plate (BD Biosciences), and cultured at 37° C for 2 h. Macrophages were used after non‐adherent cells were washed away.^20^ CFSE‐labeled target cells (5 × 10^5^) were incubated with syngeneic B6 peritoneal cavity (PerC) macrophages (5 × 10^5^) together with 10 μg/mL of either anti‐mouse SIRPα mAb or isotype‐matched control Ab for an appropriate length of time at a 1:1 or 1:2 effector‐to‐target ratio. Macrophages counterstained with APC‐labeled anti‐F4/80 and CFSE‐labeled target phagocytosis were measured by FCM analysis. Assays were repeated at least four times.

### 2.8 In vivo phagocytosis assay

CFSE‐labeled target cells were injected into the peritoneal cavity of each B6 mouse 24 h after the systemic administration of 400 μg isotype‐matched control Ab, anti‐SIRPα mAb, anti‐CD47 mAb, or a combination of anti‐SIRPα and CD47 mAbs. To eliminate the effect of anti‐CD47 mAb binding to host immune cells, anti‐CD47 mAb was selectively pre‐coated with target cells for 30 min at 37° C ex vivo*,* and then cells were washed by PBS and pelleted by centrifugation. Target cells were re‐suspended in M199 medium (Sigma‐Aldrich) and then injected into the peritoneal cavity. After 3 h, peritoneal cells were harvested and macrophages that had phagocytosed target cells could be identified as F4/80^+^ CFSE^+^ populations by FCM analysis.

### 2.9 Cell migration assay

A cell migration assay using a Transwell culture system was performed as described previously.^21^ Briefly, B6 peritoneal macrophages were labeled with PKH26 using PKH26 Fluorescent Cell Linker Kits (Sigma‐Aldrich) and incubated for 30 min at 37° Ϲ in RPMI medium together with isotype‐control Ab, anti‐SIRPα mAb, or anti‐CD47 mAb. A portion of the cell suspension (5 × 10^5^ cells in 200 μL) was then transferred to a polycarbonate filter (pore size, 3 μm; BD Falcon) in the upper compartment of the Transwell apparatus, and fresh culture medium (600 μL) was added to the lower chamber of a 24‐well plate (BD Falcon). The number of cells that migrated into the lower compartment over 24 h was then counted with a hemocytometer and expressed as a percentage of the total number of cells added to the upper compartment. Migrated cells were counted at 200× magnification in nine adjacent microscope fields for each membrane. The assay was repeated at least four times.

### 2.10 Long‐term in vivo study with liver metastasis model

CMT93 (5 × 105 cells) was suspended in Medium 199 and injected by portal vein into 9‐ to 12‐week‐old B6 mice. 24 h before tumor inoculation, 300 μg of NK1.1‐depleting antibody (PK136) and anti‐SIRPα mAb and anti‐CD47 mAb was injected by i.p. every 3 d until the day of mouse dyed.

### 2.11 Immunohistochemical analysis

Dissected colon fragments were immediately immersed in Tissue Tek OCT compound (Sakura Finetel, Torrance, CA, USA) and cryopreserved in liquid nitrogen. Immunohistochemistry was performed on 6‐μm‐thick sections incubated with primary biotinylated Abs specific for F4/80, CD47 (EPR4150(2), Abcam) followed by incubation with streptavidin‐HRP (Vector Laboratories, Burlingame, CA, USA) and detection using 3‐amino‐9‐ethylcarbazole (Sigma‐Aldrich) as a substrate. Immunoreactive cells were counted at 400× magnification and normalized against the colon tissue surface area. The sections were stained with hematoxylin and eosin (HE).

### 2.12 Immunofluorescent staining

Cell lines and mouse peritoneal macrophages were stained with PE‐conjugated anti‐mouse CD47 (miap 301) mAbs (BioLegend) or PE‐conjugated anti‐mouse CD172a mAb (BD). Microscopic images were demonstrated at 40× magnification with a fluorescent microscope (BZ‐9000 Keyence, Osaka Japan).

### 2.13 Real‐time quantitative RT‐RCR

RNA was prepared from murine cell lines using RNeasy mini Kit (QIAGEN, Germany). cDNA was prepared from RNA using QuantiTect Reverse Transcription Kit (QIAGEN). CD47 mRNA was measured by real‐time quantitative RT‐RCR, using the Rotor Gene SYBR Green PCR kit in Rotor Gene Q system (QIAGEN) according to manufacturer's instructions. The primers used in PCR are as follows: 5′ primer (5′‐CACAGTCATCGTGGTTGTTGGA‐3′) and 3′ primer (5′‐GTGATCAATATGGCAATGGTGAAAG‐3′) for mouse CD47; 5′ primer (5′‐GCGGCATCTTCAAACC‐3′) and 3′ primer (5′‐TGCCGTGTGAACCATG‐3′) for β2‐Microglobulin (eurofins Genomics) were used as loading control.

### 2.14 In vitro phagocytosis assay using human reticuloendothelial macrophages

Human reticuloendothelial macrophages were isolated as described previously.^14^ In brief, the mononuclear cells were isolated from the perfusion effluents of liver allografts for clinical liver transplantation by gradient centrifugation with Separate‐L (Muto Pure Chemicals Co., Ltd, Tokyo, Japan). To prepare macrophages, cells were plated in a 24‐well plate (BD Biosciences) and cultured at 37° Ϲ for 2 h. Macrophages were used after non‐adherent cells were washed away. The purity of CD14^+^ macrophages was confirmed by FCM analysis immunofluorescence using FACS Calibur (Becton Dickinson, Mountain View, CA, USA). More than 95% of the cells demonstrated positivity for the CD14 antigen. Without any pre‐culture, freshly isolated human RE macrophages were immediately subjected to the phagocytosis assays. Anti‐human SIRPα mAb (4C7) and anti‐human CD47 mAb (B6H12) were used for human phagocytosis assay using CFSE‐labeled human hepatoma cell line (Huh7) as targets. Huh7 cells were purchased from The Japanese Cancer Research Resources Bank and were maintained in 10% RPMI. Ethical approval for this study was obtained from the Ethics Committee at The Hiroshima University Hospital. Informed consent was obtained from all donors for participation in this study.

### 2.15 Statistical analysis

Data are presented as the means ± SD and were analyzed by the Mann–Whitney *U*‐test. Survival curves were generated using the Kaplan–Meier method and compared between different groups by log‐rank tests. A *P*‐value of <0.05 was considered statistically significant. All statistical analyses were performed using SPSS software (version 22; IBM Corp., Armonk, NY).

## 3 RESULTS

### 3.1 Knockdown of CD47 expression increases gastroenterological tumor cell susceptibility to peritoneal macrophage‐mediated phagocytosis

**Figure 1:**
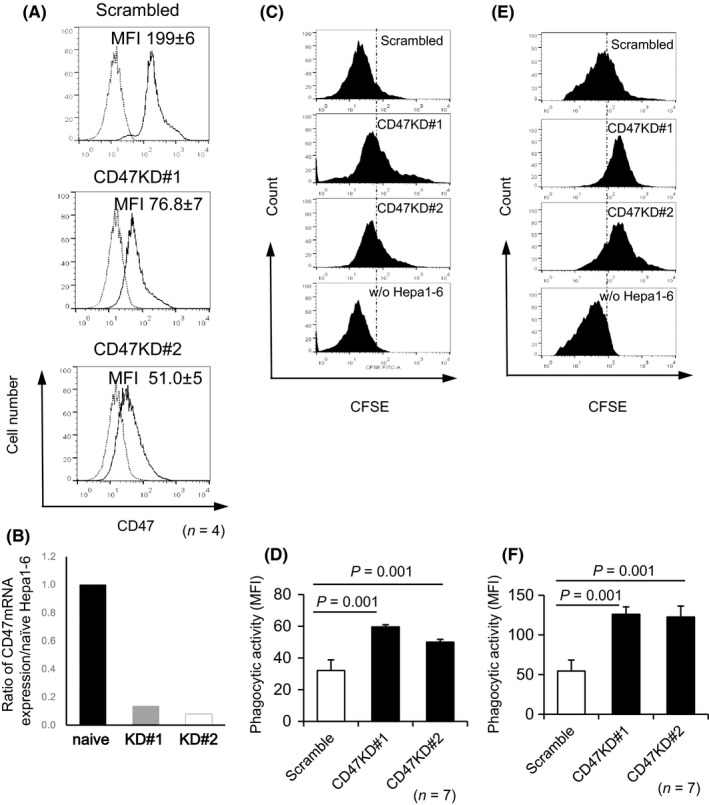
Effect of CD47 knockdown on macrophage phagocytosis of Hepa1‐6 cells in vitro and in vivo. A, Representative histograms obtained by FCM analysis for scrambled Hepa1‐6 cells (Scrambled) or CD47‐knockdown (CD47KD) Hepa1‐6 cells treated with shRNA#1 or shRNA#2 (CD47KD #1, CD47KD#2). MFI, mean fluorescent intensity. CD47 expression was detected by FCM using anti‐CD47 (solid line) and isotype‐control (dotted line) Abs. B, CD47 mRNA level by RT‐qPCR for knockdown proportion of CD47 in Hepa1‐6 cells. ß2‐microglobulin was used as loading control. Representative FCM data of macrophage phagocytosis for the same populations of cells in vitro (C) or in vivo (E). Histogram calculated by determining the number of F4/80 positive macrophages that phagocytosed CFSE‐stained Hepa1‐6 cells. From top to bottom, scrambled Hepa1‐6 cells, CD47KD#1 Hepa1‐6 cells, CD47KD#2 Hepa1‐6 cells, and macrophages without Hepa1‐6 cells. D, F, Phagocytic indices of CD47KD Hepa1‐6 cells (black bars) and scrambled Hepa1‐6 cells (white bar) from in vitro (D) or in vivo (F) phagocytosis assay (*n* = 7)

To investigate the significance of CD47‐SIRPα interactions in macrophage action against gastroenterological tumors, we induced lentiviral‐mediated CD47 protein expression knockdown in Hepa1‐6 hepatoma and CMT93 colon carcinoma cells originating from B6 mice. FCM analysis revealed that CD47 expression levels in CD47‐knockdown (KD) Hepa1‐6 cell lines (CD47KD#1 and CD47KD#2 Hepa1‐6 cells) were reduced to >70% that of control vector‐transfected Hepa1‐6 cells (scrambled Hepa1‐6 cells) (Figure 1A). The CD47 mRNA expression was quantified by using real‐time RT‐qPCR for demonstrating CD47 knockdown in Hepa1‐6 hepatoma cells. CD47 mRNA levels of CD47KD#1 and CD47KD#2 Hepa1‐6 cell lines were decreased when compared with naïve Hepa1‐6 cells (Figure 1B). The decrease of CD47 was also confirmed by fluorescent immunostaining (Figure S2A). CFSE‐labeled tumor cells were used as targets, and peritoneal macrophages isolated from B6 mice were used as effectors in an in vitro phagocytosis assay in which the macrophages engulfing target cells could be identified by FCM. Both CD47KD#1 and CD47KD#2 Hepa1‐6 cells were significantly more susceptible to macrophage‐mediated phagocytosis compared to scrambled Hepa1‐6 cells (*P *=* *0.001 for both) (Figure 1C,D). These results were consistent with those of the in vivo phagocytosis assay, wherein CFSE‐labeled CD47KD#1 and CD47KD#2 Hepa1‐6 cells were more susceptible to phagocytosis than scrambled Hepa1‐6 cells following intraperitoneal injection into B6 mice (*P *=* *0.001 for both) (Figure 1E,F). FCM analysis revealed that CD47KD#1 CMT93 cell CD47 expression levels were reduced to >10% that of control vector‐transfected CMT93 cells (scrambled CMT93 cells), whereas CD47KD#2 CMT93 cells sustained 50% of control CD47 expression levels (Figure S1A). CD47 mRNA levels of CD47KD#1 and CD47KD#2 CMT93 cell lines were decreased when compared with naïve CMT93 cells (Figure S1B). The decrease of CD47 was also confirmed by fluorescent immunostaining (Figure S2B). In vivo phagocytosis assays also revealed that CD47KD CMT93 cells were susceptible to macrophage phagocytosis in a CD47 expression level‐dependent manner (*P *=* *0.034) (Figure S1C and D).

### 3.2 Treatment with anti‐SIRPα mAb increases gastroenterological tumor cell susceptibility to macrophage‐mediated phagocytosis

**Figure 2:**
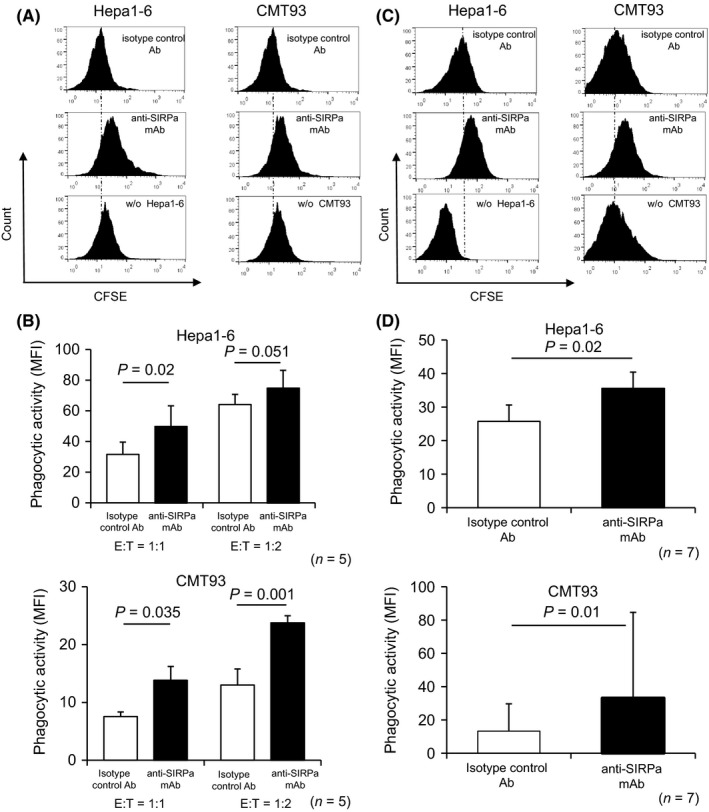
Effect of anti‐SIRPα mAb treatment on macrophage phagocytosis of Hepa1‐6 and CMT93 cells in vitro and in vivo. A, C, Representative histograms showing in vitro (A) or in vivo (C) phagocytic activity of peritoneal macrophages toward Hepa1‐6 and CMT93 cells treated with isotype‐matched control Ab or anti‐SIRPα mAb. From top to bottom, scrambled cells, isotype‐matched control Ab treatment, anti‐SIRPα mAb treatment, and macrophages without target cells. B, D, In vitro (B) or in vivo (D) phagocytic activity of peritoneal macrophages toward Hepa1‐6 and CMT93 cells treated with isotype‐matched control Ab or anti‐SIRPα mAbs, as determined by mean fluorescent intensity (MFI) (*n* = 5 (B), *n* = 7 (D). E:T  =  effector‐to‐target ratio

**Figure 3:**
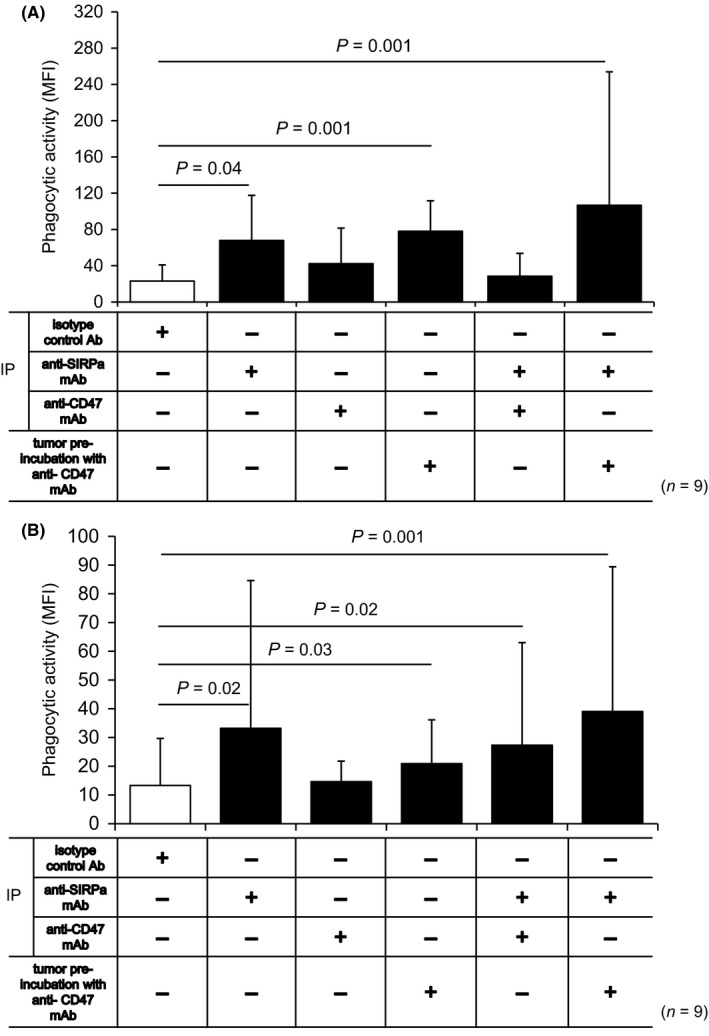
Effect of systemic treatment with anti‐SIRPα and/or anti‐CD47 mAbs on Hepa1‐6 and CMT93 cancer cells. A, B, Phagocytic activity of macrophages against Hepa1‐6 (A) or CMT93 (B) cells systemically treated with isotype‐matched control Ab, anti‐SIRPα mAb, and/or anti‐CD47 mAb and against Hepa1‐6 cells pre‐inoculated with anti‐CD47 mAb (*n* = 9)

We next investigated the therapeutic impact of mAbs blocking CD47 and/or SIRPα on gastroenterological tumors in immunocompetent mouse models equipped with syngeneic immune responses between cancer and immune cells. Immunohistochemistry revealed that the expression of CD47 and SIRPα was detected in peritoneal macrophages, whereas SIRPα expression was not detected in both Hepa1‐6 and CMT93 (Figure S2A‐C). Pre‐incubation of anti‐CD47 mAb resulted in the complete cover of CD47 expression with the corresponding mAb on both Hepa1‐6 and CMT93 (Figure S2A,B). In the in vitro phagocytosis assay, adding anti‐SIRPα mAb resulted in a significant increase in B6 background‐Hepa1‐6 and CMT93 cell susceptibility to peritoneal macrophage‐mediated phagocytosis from B6 mice in a 1:1 (*P *=* *0.02, Hepa1‐6; *P *=* *0.035, CMT93) and 1:2 (*P *=* *0.051, Hepa1‐6; *P *=* *0.001, CMT93) effector‐to‐target ratio (Figure 2A,B). Similarly, according to in vivo phagocytosis assays, systemic anti‐SIRPα mAb administration by i.p. significantly promoted Hepa1‐6 and CMT93 cell macrophage phagocytosis in the peritoneal cavity of B6 mice (*P *=* *0.02 and *P *=* *0.01, respectively) (Figure 2C,D). As neither Hepa1‐6 nor CMT93 cells express surface SIRPα (Figure S2A,B), anti‐SIRPα mAb exclusively targeted peritoneal macrophages in these models. Notably, the similarly administered anti‐CD47 mAb did not promote Hepa1‐6 and CMT93 cell macrophage phagocytosis in the in vivo phagocytosis assay, and combined anti‐SIRPα and anti‐CD47 mAb administration by i.p. did not lead to any synergistic macrophage phagocytosis effects (Figure 3A,B). Since IgG2 subclass of anti‐CD47 mAb was used in this study, biding anti‐CD47 mAb to CD47 on inoculated cancer cells theoretically promotes opsonizing effects via Fcγ/Fcγ receptor pathway. Consistently, Hapa1‐6 and CMT93 cells incubated with anti‐CD47 mAb prior to inoculation were further susceptible to macrophage‐mediated phagocytosis in mice treated with anti‐SIRPα mAb inhibiting CD47‐SIRPα “don't eat me” signal, when compared with non‐manipulated those tumor cells, although the differences did not reach the statistical significance (Figure 3).

**Figure 4:**
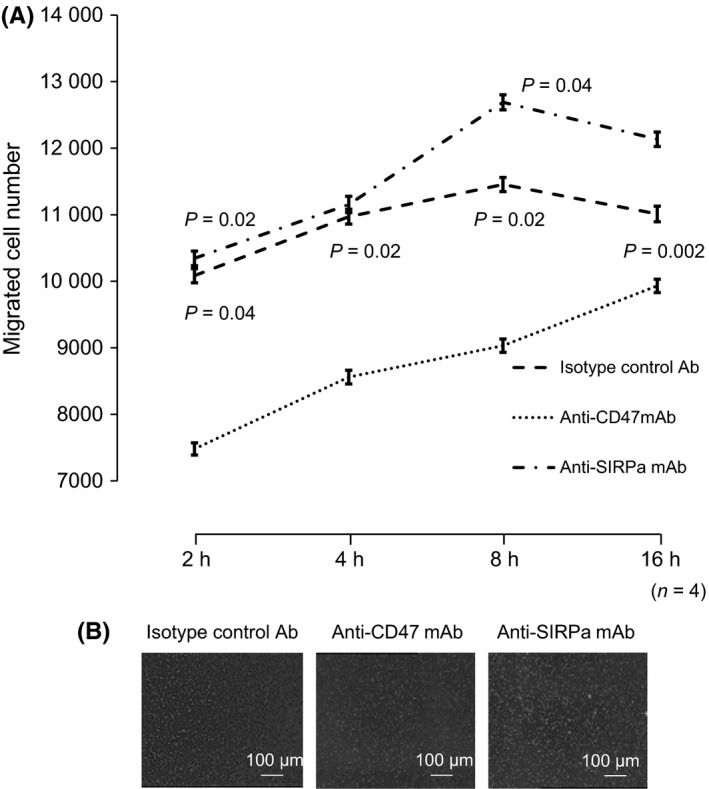
Effect of anti‐CD47 mAb and anti‐SIRPα mAb on macrophage migration. A, Migration of mouse macrophages treated with the indicated mAb (*n* = 4). The number of migrated cells was significantly lower following anti‐CD47 mAb treatment than that following isotype‐control Ab treatment at 2‐16 h. B, Representative images of mouse macrophage migration following treatment with isotype‐control Ab, anti‐CD47 mAb, or anti‐SIRPα mAb at 8 h

A previous finding demonstrating the role of CD47 in polymorphonuclear neutrophil transmigration across tissue cells, and the extracellular matrix raised the question of whether CD47 may also mediate macrophage transmigration/chemotaxis.^21^, ^22^ To address this possibility, freshly isolated B6 mouse peritoneal macrophages were cultured in Transwell tissue culture plate upper compartments in the presence/absence of either anti‐CD47 or anti‐SIRPα mAb for 24 h. Macrophage migration through the membrane was significantly inhibited in the presence of anti‐CD47 mAb (*P *=* *0.02 at 2 h, *P *=* *0.04 at 8 h vs isotype‐control group; *P *=* *0.04 at 2 h, *P *=* *0.02 at 4 h, *P *=* *0.02 at 8 h, *P *=* *0.002 at 16 h vs anti‐SIRPα mAb group), whereas anti‐SIRPα mAb did not affect transmigration through the membrane, suggesting that signaling through CD47, rather than SIRPα, may be involved in macrophage transmigration/chemotaxis (Figure 4A,B). Hence, it is likely that in vivo systemic anti‐CD47 mAb administration will lead to less frequent contact with macrophages and tumor cells, limiting its effect on macrophage phagocytosis.

**Figure 5:**
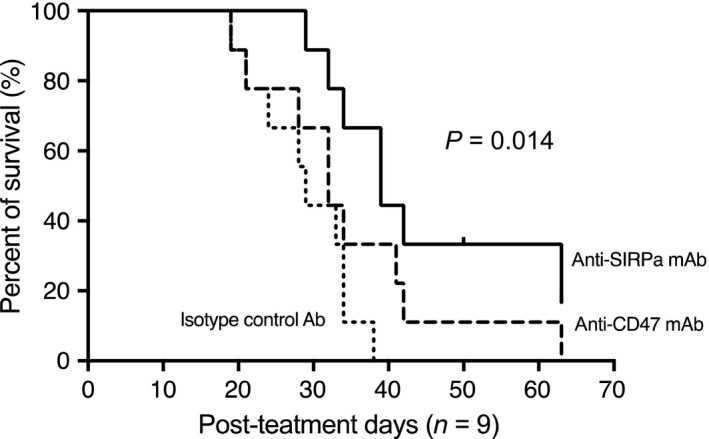
Kaplan–Meier analysis demonstrated that anti‐SIRPα mAb therapy significantly prolonged the prognosis in syngeneic mouse colorectal liver metastasis models compared with anti‐CD47mAb and isotype‐matched control Ab treatments (*n* = 9) [Correction added on 5 October 2018, after first online publication: Figure 5 has been amended due to a missing curve.]

We further evaluated the effects of anti‐CD47 and anti‐SIRPα mAbs by inoculating Hepa1‐6 and CMT93 via the portal vein in B6 mice as a long‐term in vivo study. Those cancer cells were not able to be constantly engrafted in untreated syngeneic B6 mice, probably reflecting their vigorous innate immune responses. We found that depletion of NK and NKT cells by using NK1.1 mAb in mice achieved liver metastasis of colorectal cancer cell CMT93 but could not do so with HCC Hepa1‐6. In the colorectal liver metastasis model with CMT93, the treatment with anti‐SIRPα mAb significantly prolonged the survival of mice inoculated with CMT93, but that with anti‐CD47 mAb did not (Figure 5).

### 3.3 Anti‐SIRPα mAb therapy inhibits tumor progression in a sporadic colon cancer mouse model with conditional mutations in APC

To investigate anti‐SIRPα mAb therapy inhibitory effects on sporadic solid neoplastic tumor growth, we used a conditional APC knockout *CPC‐APC* mouse model, in which colon‐preferential gene targeted mice recapitulate human colorectal cancer.^16^ We assessed anti‐SIRPα mAb treatment effects on tumor growth in the colon and small intestine of B6 background‐*CPC‐APC* mice that received anti‐SIRPα mAb weekly from 8 weeks of age until death. Isotype‐matched irrelevant Ab was administered in control mice.

**Figure 6:**
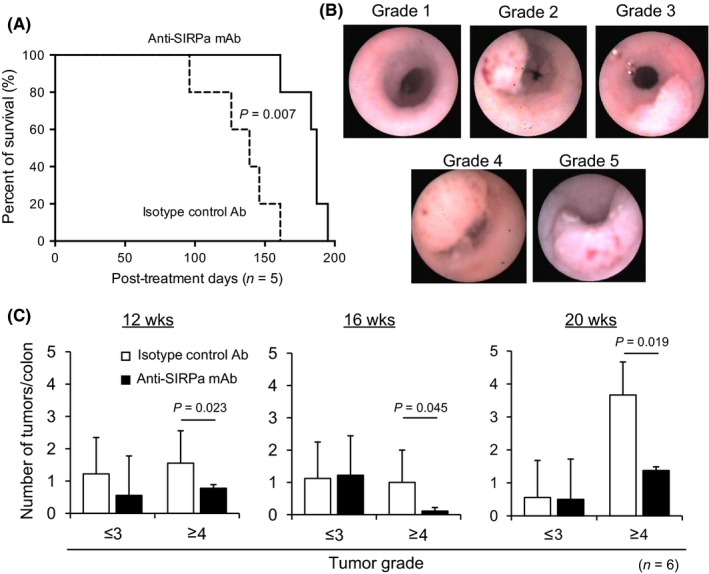
Effect of anti‐SIRPα mAb on tumor progression in *CPC‐APC* mice. A, Kaplan‐Meier analysis of long‐term survival of *CPC‐APC* mice treated with anti‐SIRPα mAb (solid line) or isotype‐control Ab (dashed line) (*n* = 5). B, Representative images of mouse colonoscopies showing each tumor grade in *CPC‐APC* mice. C, Tumor number sorted by grade in *CPC‐APC* mice treated with isotype‐matched control Ab or anti‐SIRPα mAb for the indicated times following treatment initiation (*n* = 6)

**Figure 7:**
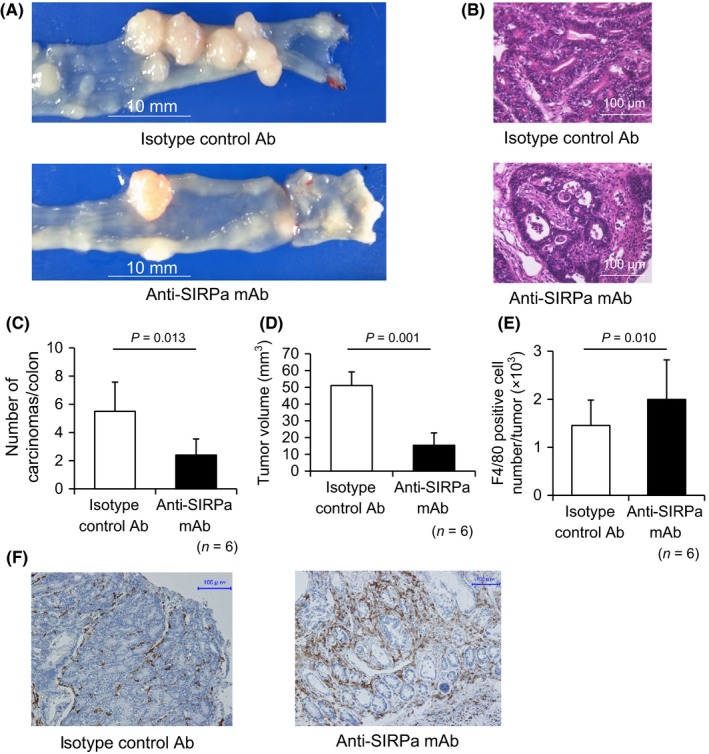
Effect of anti‐SIRPα mAb on tumor growth and infiltration by F4/80‐positive macrophages in colons of *CPC‐APC* mice. A, Representative image of sporadic colon tumor growth and occurrence in colons of *CPC‐APC* mice after treatment with anti‐SIRPα mAb or isotype‐matched control Ab. B, HE staining of colon tumor growth in *CPC‐APC* mice after treatment with anti‐SIRPα mAb or isotype‐matched control Ab (16 × magnification). C–E, Number of colon carcinomas (C), tumor volume (D), and tumor infiltration of F4/80‐positive macrophages in tumors (E) of *CPC‐APC* mice treated with anti‐SIRPα mAb or isotype‐matched control Ab killed at 20 week after the initiation of treatment (*n* = 6). F, Immunohistochemical analysis of F4/80‐positive macrophage tumor infiltration in tumors of *CPC‐APC* mice treated with anti‐SIRPα mAb or isotype‐matched control Ab (40 × magnification)

For evaluation of tumor number and size, mouse colonoscopies were performed using a grading system according to tumor circumference and the area of the colon that it occupied. In untreated *CPC‐APC* mice, the adenoma developed to type 1 cancer predominantly in the left colon from 9 weeks of age. Anti‐SIRPα mAb treatment significantly improved *CPC‐APC* mouse survival compared with that of isotype‐matched control Ab (*P *=* *0.007) (Figure 6A). Colonoscopy revealed that the numbers of tumors progressing to a grade higher than 4 at 12, 16, and 20 weeks of age were strongly suppressed by anti‐SIRPα mAb treatment (*P *=* *0.023 at 12 weeks, *P *=* *0.045 at 16 weeks, *P *=* *0.019 at 20 weeks) (Figure 6B,C). At 20 weeks of age, experimental mice were euthanized to harvest the entire colon for histological studies. Colon tumors were macroscopically counted (Figure 7A) and microscopically confirmed as adenocarcinomas (Figure 7B). Colon adenocarcinomas exhibited over‐expression of CD47 (Figure S3). At 20 weeks from treatment initiation, tumor number and volume per colon were significantly reduced in mice treated with anti‐SIRPα mAb when compared with those in mice treated with isotype‐matched control Ab (*P *=* *0.013, *P *=* *0.001) (Figure 7C,D). The average number of F4/80‐positive cells (predominantly macrophages) infiltrating each tumor was also significantly higher in anti‐SIRPα mAb‐treated mice (*P *=* *0.010) (Figure 7E,F).

### 3.4 Anti‐SIRPα mAb treatment enhances phagocytic activity of human reticuloendothelial macrophages against human hepatoma cells

**Figure 8:**
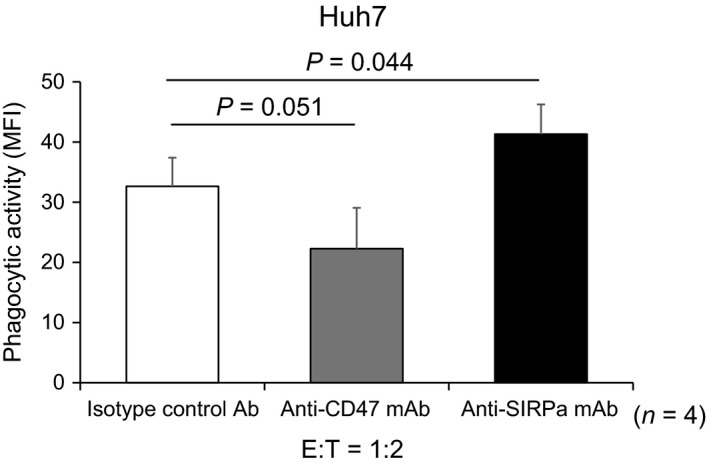
*In vitro* phagocytosis assay, human LMNC was significantly enhanced phagocytic activity toward Huh7 compared with anti‐CD47mAb and isotype‐matched control Ab (*n* = 4)

We carried out an additional study of in vitro phagocytosis assay using human hepatoma cell line (Huh7), and human reticuloendothelial macrophages obtained from liver allograft perfusate collected at liver transplantation, as a further clinically relevant model. Consistent with the results from mouse studies, the treatment with anti‐SIRPα mAb significantly enhanced phagocytic activity of human macrophages against human hepatoma cell line, compared with that with isotype‐matched control Ab, whereas that with nti‐CD47mAb rather reduced (Figure 8).

## 4 DISCUSSION

CD47 functions as a phagocytosis inhibitor through ligation of SIRPα expressed on phagocytes, leading to tyrosine phosphatase activation and inhibition of myosin accumulation at the phagocytic synapse submembrane assembly site.^23^ CD47 loss leads to homeostatic phagocytosis of aged or damaged cells.^5^, ^7^, ^24^ We have demonstrated that interspecies CD47 incompatibility in xenotransplantation leads to SIRPα‐mediated “don't eat me” signal absence, resulting in the rejection of xenogeneic cells by human macrophages.^14^, ^20^ Whereas macrophages can be activated by pro‐phagocytic signaling pathways through activating receptors such as Fcγ and complement receptors, their phagocytic activity is also controlled by immune inhibitory receptor the signal strength.^25^ Additionally, lectin‐mediated carbohydrate binding may provide activating signals to macrophages without opsonization.^26^ We have also demonstrated that a CD47‐SIRPα inhibitory signal induced by genomic manipulation overrides such an activating signal delivered to macrophages by xeno‐antigens. Increased cell surface heterophilic carbohydrate antigen expression can be targeted by macrophages, such as Thomsen‐Friedenreich antigen (Galß1‐3GalNAcα‐), a common feature in malignant and pre‐malignant epithelia.^27^ Hence, it is likely that CD47‐SIRPα signaling may also override the activating signals delivered to macrophages by cancer antigens. Emerging evidence indicates that in various neoplastic cells including solid cancers, CD47 expression is required to avoid innate immune surveillance and elimination by phagocytosis. Increased CD47 expression has been reported to enable cancer cells to evade macrophage phagocytosis.^8^, ^9^, ^11^, ^12^, ^28^, ^29^ Here, we demonstrated that the CD47‐SIRPα interaction inhibits macrophage‐mediated phagocytosis, even in gastroenterological tumors.

CD47 is expressed on multiple human tumor types including acute and chronic myeloid leukemia, acute lymphoblastic leukemia, non‐Hodgkin's lymphoma, multiple myeloma, bladder cancer, and other solid tumors.^8^, ^9^, ^10^, ^12^, ^30^, ^31^ Whereas CD47 is ubiquitously expressed at low levels on normal cells, multiple tumors express increased CD47 levels compared to their normal cell counterparts.^10^, ^29^ Although it has been recently demonstrated that hypoxia‐inducible factor 1 directly activates *CD47* gene transcription in hypoxic solid cancer cells,^32^ the molecular mechanisms regulating CD47 expression in cancer cells have not been fully determined. Nevertheless, numerous studies have demonstrated that targeting of CD47 produces noticeable effects on tumor growth inhibition and metastasis prevention of various human cancer types in immunodeficient mouse xenotransplantation models.^9^, ^10^, ^11^, ^33^ Recently, studies using syngeneic immunocompetent mouse models have shown that anti‐CD47 mAb treatment not only enables macrophage phagocytosis of cancer but can also initiate an anti‐tumor cytotoxic T‐cell immune response.^34^, ^35^, ^36^, ^37^ Although the reciprocal effects of anti‐CD47 therapy on innate and adaptive anti‐tumor immune responses have been well investigated in these studies, including following anti‐CD47 mAb or CD47 translation‐blocking antisense morpholino intratumoral injection, the net influence of systemic anti‐CD47 mAb administration on either macrophage phagocytotic activity or other intrinsic CD47‐expressing cell activity has not been intensively investigated.

Consistent with these previous findings, which suggest that CD47 targeting represents a potentially effective therapeutic strategy, intraperitoneally administered anti‐CD47 mAb did not promote macrophage phagocytosis of gastrointestinal cancer cells in our syngeneic immunocompetent mouse model, although cancer cells incubated with anti‐CD47 mAb prior to inoculation were susceptible to macrophage‐mediated phagocytosis. As CD47 also serves as a membrane‐associated glycoprotein that suppresses immune cell function, anti‐CD47 mAb binding with host immune cells may influence their intrinsic functions.^21^, ^22^ Notably, murine dendritic cell chemotaxis is significantly reduced by anti‐CD47 mAb treatment.^38^ Similarly, CD47 has a role in polymorphonuclear neutrophil transmigration across tissue cells and the extracellular matrix;^22^ thus, we hypothesized that CD47 on macrophages also mediates their transmigration/chemotaxis.^21^, ^22^, ^39^, ^40^ Consistent with this assumption, we observed that anti‐CD47 mAb treatment markedly inhibited macrophage transmigration through a Transwell membrane in vitro. Hence, in vivo systemic anti‐CD47 mAb administration likely impaired macrophage transmigration/chemotaxis, resulting in no net effect on phagocytosis.

Rather than targeting CD47, we demonstrated the suppressive effects of anti‐SIRPα mAbs on gastrointestinal cancer cell ability to evade macrophage phagocytosis in syngeneic immunocompetent mouse models. Our findings from the *CPC‐APC* mouse model experiments provide direct evidence that SIRPα targeting contributes to tumorigenesis suppression and prolongs survival. Potential limitations of anti‐CD47 mAb treatment include the observation that direct CD47 targeting impairs macrophage migration and that CD47 expression on normal cells may create sites of toxicity or sequester CD47 targeting agents by competitive adsorption. SIRPα targeting by mAb may overcome these limitations. Another benefit of targeting SIRPα on phagocytes rather than CD47 on tumors is the potential for synergy with tumor‐specific mAbs to increase phagocytosis and enhance the anti‐tumor response. Currently, several targeted therapies involving mAb binding to tumor‐associated antigens have been developed as promising cancer treatments. Such mAbs can bridge immune effector cells with tumor cells, resulting in antibody‐dependent cytotoxicity. In addition, increasing evidence has identified macrophages as prominent effector cells and antibody‐dependent cell phagocytosis induction as a primary mechanism of action mediated by mAbs.^41^, ^42^, ^43^ Hence, potential synergistic anti‐tumor effects involving SIRPα targeting agents and tumor‐specific mAbs should be investigated in future experiments.

Pan YF et al. have demonstrated that soluble factors derived from hepatomas trigger transient activation of newly recruited macrophages and reduce SIRPα expression, thereby inducing these cells to produce a large amount of cytokines, in turn leading to the down‐expression of SIRPα on macrophages and ultimately create an inflammatory environment supporting tumor progression.^44^ Their findings suggest that there is a fine‐tuned collaborative action between SIRPα expression on macrophages and tumor progression. SIRPα is tyrosine phosphorylated and sequestrates SHP2 from IKKß to PI3K regulatory subunit PI3Kp85, resulting in affecting PI3K‐Akt and NF‐κB pathways in the tumor microenvironment. Therefore, it is likely that administration of anti‐CD47 and/or anti‐SIRPα mAb interferes Akt and NF‐κB activation in macrophages, influencing macrophages migration, survival, cytokines production, and/or angiogenesis in tumor sites.^44^ We have demonstrated that anti‐SIRPα mAb promotes phagocytic activity of macrophages not only against hepatoma but also against colon carcinoma cells, but anti‐CD47 mAb does not. It remains to be elucidated whether anti‐SIRPα mAb causes functional alteration of tumoricidal macrophages or not.

In conclusion, we have demonstrated that anti‐SIRPα mAb exhibits enhanced macrophage phagocytic activity and marked anti‐tumor effects in immunocompetent syngeneic mouse models. Considering the negative effect of anti‐CD47 mAb on host immune cells, the augmentation of macrophage phagocytic activity by anti‐SIRPα mAb may constitute an effective treatment for human gastrointestinal tumors.

## DISCLOSURES

Funding: This work was supported by a JSPS KAKENHI Grant Number JP23249064, a grant from the Research Program on Hepatitis from Japan Agency for Medical Research and Development (AMED; JP15fk0210016 h003 and JP16fk0210107).

Conflict of interest: The authors declare no conflict of interests.

Ethical statements: All animal protocols described in this study were performed in accordance with the Guide for the Care and Use of Laboratory Animals and the local committee for animal experiments. The experimental protocol was approved by the Ethics Review Committee for Animal Experimentation of the Graduate School of Biomedical Sciences, Hiroshima University (A13‐70).
