# Supplementary material for: Signal regulatory protein alpha blockade potentiates tumoricidal effects of macrophages on gastroenterological neoplastic cells in syngeneic immunocompetent mice
Source: Ann Gastroenterol Surg. 2018 Sep 10;2(6):451–62. doi: 10.1002/ags3.12205 (PMC6236110; doi:10.1002/ags3.12205)
Supplement: Supplementary file 1 [file AGS3-2-451-s001.pptx]

## Slide 1
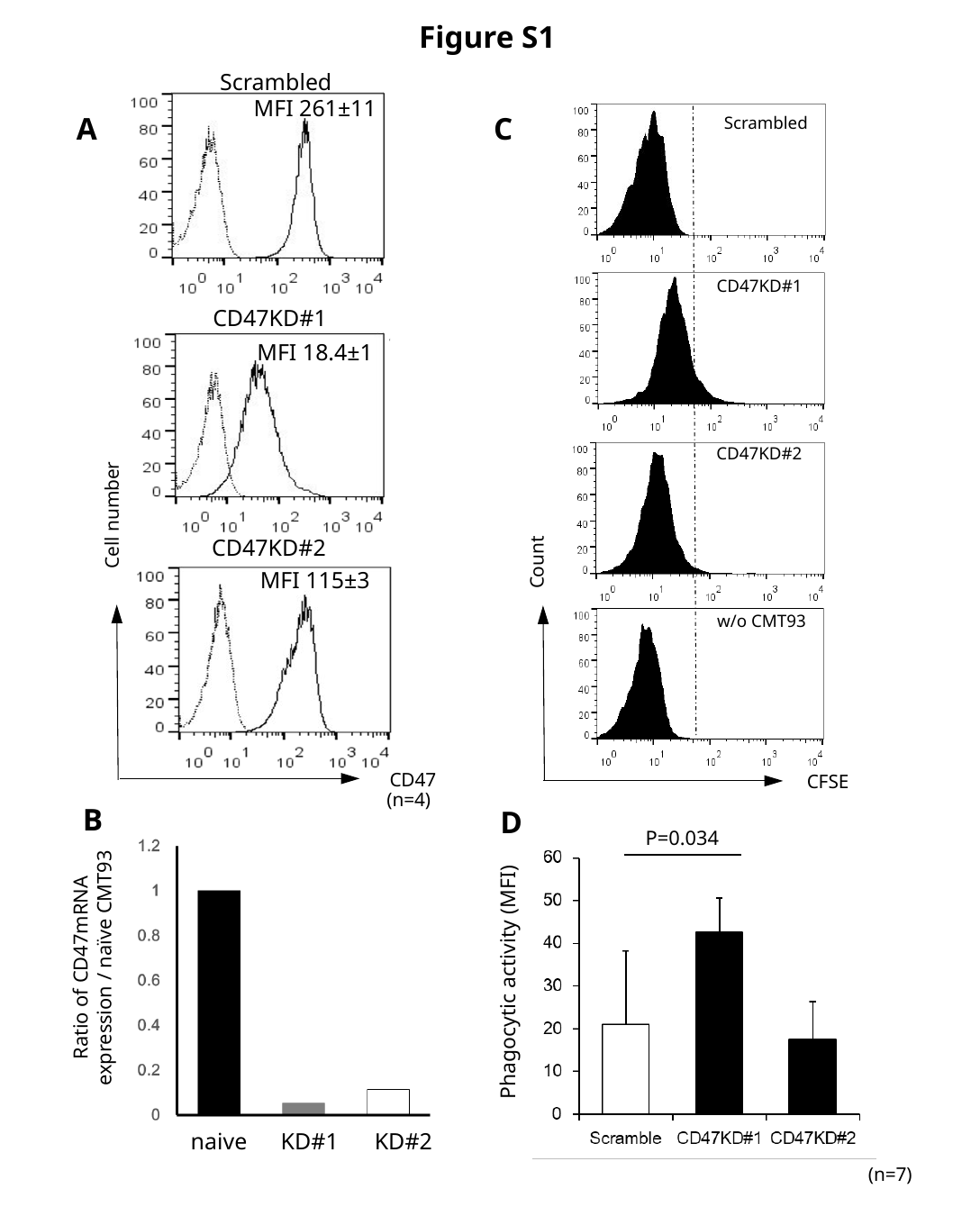

Figure S1
Scrambled
MFI 261±11
A
C
Scrambled
CD47KD#1
CD47KD#1
MFI 18.4±1
CD47KD#2
Cell number
Count
CD47KD#2
MFI 115±3
w/o CMT93
CD47
CFSE
 (n=4)
B
D
P=0.034
Phagocytic activity (MFI)
Ratio of CD47mRNA expression / naïve CMT93
KD#2
KD#1
naive
 (n=7)
