# Supplementary material for: Signal regulatory protein alpha blockade potentiates tumoricidal effects of macrophages on gastroenterological neoplastic cells in syngeneic immunocompetent mice
Source: Ann Gastroenterol Surg. 2018 Sep 10;2(6):451–62. doi: 10.1002/ags3.12205 (PMC6236110; doi:10.1002/ags3.12205)
Supplement: Supplementary file 2 [file AGS3-2-451-s002.pptx]

## Slide 1
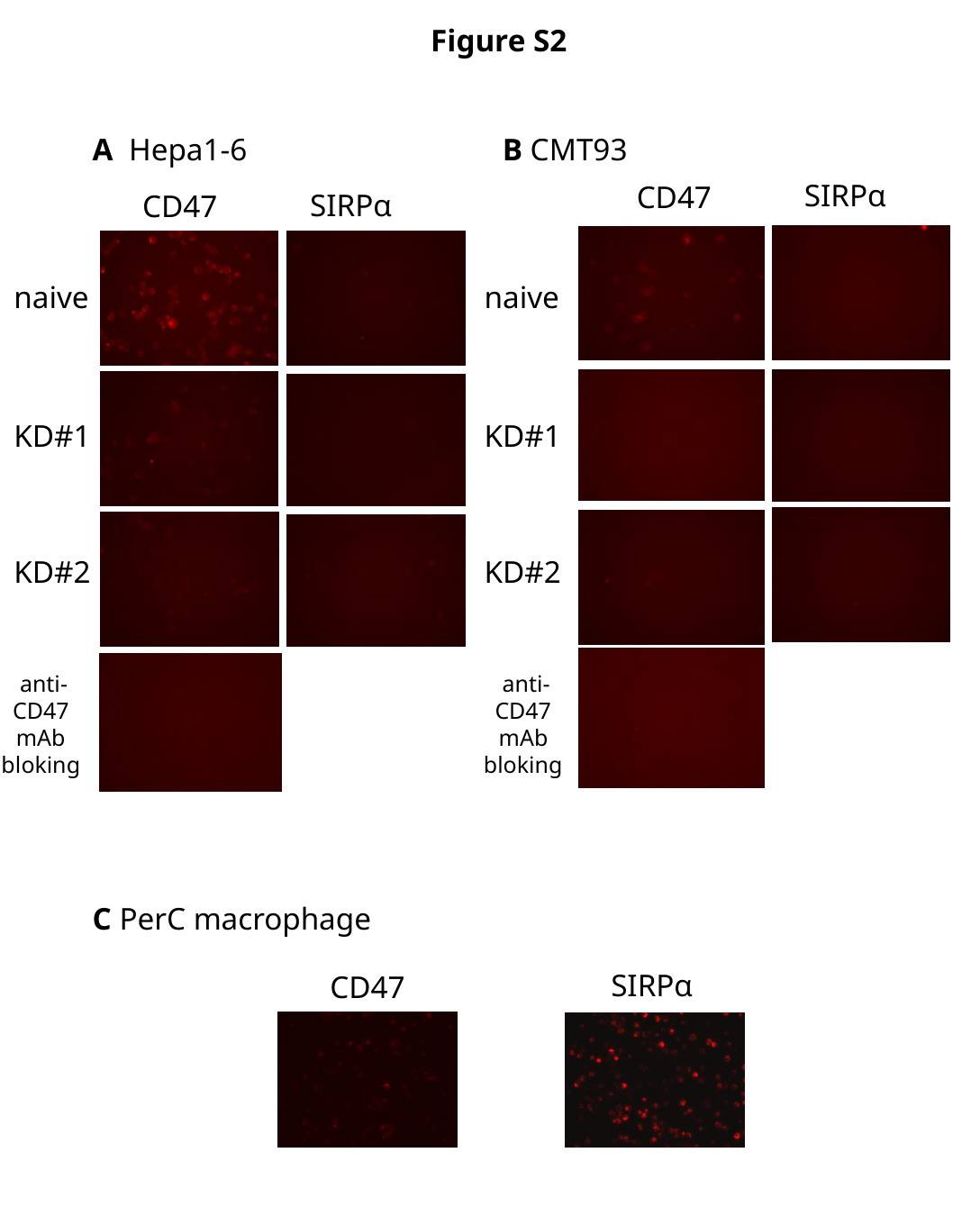

Figure S2
A Hepa1-6
B CMT93
SIRPα
CD47
SIRPα
CD47
 naive
 naive
 KD#1
 KD#1
 KD#2
 KD#2
 anti-CD47 mAb bloking
 anti-CD47 mAb bloking
C PerC macrophage
SIRPα
CD47
