# Supplementary figures and images for: Signal regulatory protein alpha blockade potentiates tumoricidal effects of macrophages on gastroenterological neoplastic cells in syngeneic immunocompetent mice
Source: Ann Gastroenterol Surg. 2018 Sep 10;2(6):451–62. doi: 10.1002/ags3.12205 (PMC6236110; doi:10.1002/ags3.12205)

## Slide 1
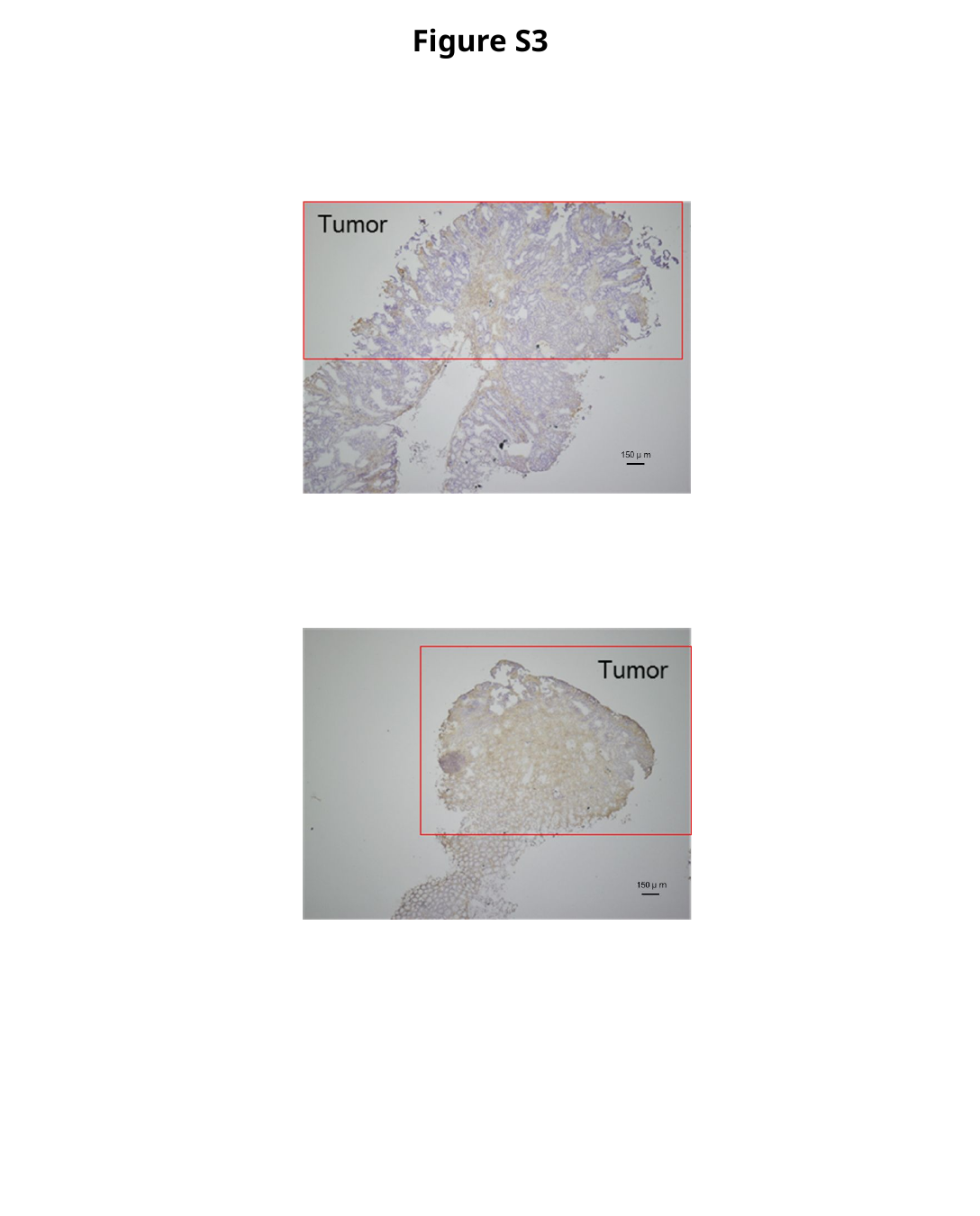

Figure S3

Supplement: Supplementary file 3 [file AGS3-2-451-s003.pptx]
