# Supplementary material for: Signal regulatory protein alpha blockade potentiates tumoricidal effects of macrophages on gastroenterological neoplastic cells in syngeneic immunocompetent mice
Source: Ann Gastroenterol Surg. 2018 Sep 10;2(6):451–62. doi: 10.1002/ags3.12205 (PMC6236110; doi:10.1002/ags3.12205)
Supplement: Supplementary file 4 [file AGS3-2-451-s004.pptx]

## Slide 1
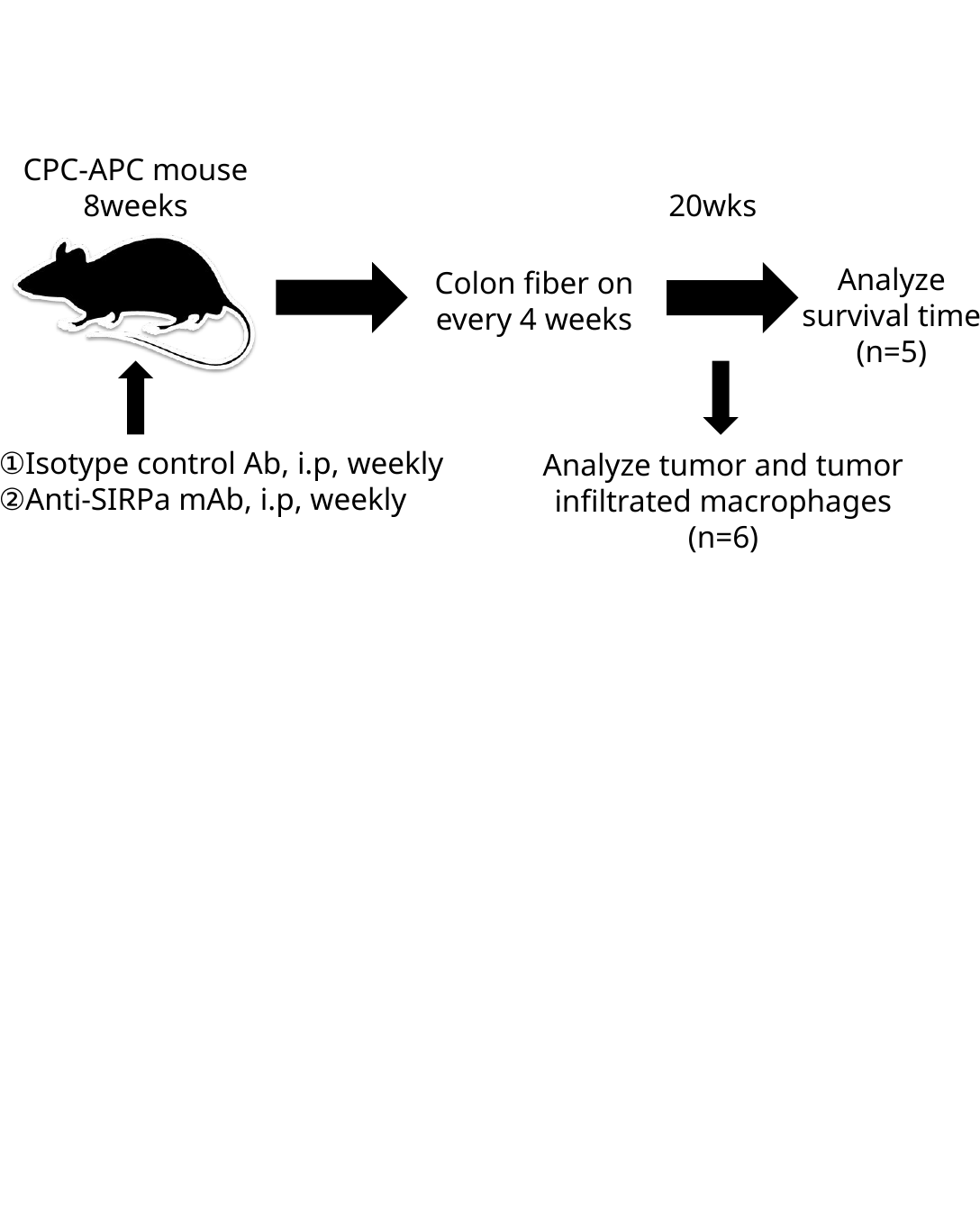

CPC-APC mouse
8weeks
20wks
Analyze survival time
(n=5)
Colon fiber on every 4 weeks
①Isotype control Ab, i.p, weekly
②Anti-SIRPa mAb, i.p, weekly
Analyze tumor and tumor infiltrated macrophages (n=6)
