# Supplementary material for: Signal regulatory protein alpha blockade potentiates tumoricidal effects of macrophages on gastroenterological neoplastic cells in syngeneic immunocompetent mice
Source: Ann Gastroenterol Surg. 2018 Sep 10;2(6):451–62. doi: 10.1002/ags3.12205 (PMC6236110; doi:10.1002/ags3.12205)
Supplement: Supplementary file 5 [file AGS3-2-451-s005.doc]

**SUPPORTING INFORMATION**

**Signal regulatory protein alpha blockade potentiates tumoricidal effects of macrophages on gastroenterological neoplastic cells in syngeneic immunocompetent mice**

Tomoyuki Abe^1^ │ Yuka Tanaka^1^ │ Piao Jinlian^1^ │ Naoki Tanimine^1^ │ Naohide Oue^2^ │ Takao Hinoi^1^ │ Noel Verjan Garcia^3^ │ Masayuki Miyasaka^4,5^ │ Takashi Matozaki^6^ │ Wataru Yasui^2^ │ Hideki Ohdan^1^

**SUPPLEMENTARY MATERIALS AND METHODS**

**Immunohistochemistry**

CD47 protein expression was evaluated on CPC-APC tumor from optimal cutting temperature compound (OCT)-embedded fresh frozen tissue. OCT-embedded frozen tissue was sectioned into-5-μm thick sections. Tissue was then blocked with 10% rabbit serum and probed with anti CD47 antibody (EPR4150(2)) or IgG-control antibody overnight at 4ºC. Samples were stained with rabbit anti-mouse Ig secondary antibody conjugated with HRP for 1h, followed by.

**SUPPLEMENTARY FIGURE LEGENDS**

**SUPPLEMENTARY FIGURE S1.** Effect of CD47 knockdown on macrophage phagocytosis of CMT93 cells *in vivo*. (A) Representative histograms obtained by FCM analysis for scrambled CMT93 cells (Scrambled) or CD47-knockdown (CD47KD) CMT93 cells treated with shRNA#1 or shRNA#2 (CD47KD#1, CD47KD#2). MFI = mean fluorescent intensity. CD47 expression was detected by FCM using anti-CD47 (solid line) and isotype-control (dotted line) Ab. (B) CD47 mRNA level by RT-qPCR for knockdown proportion of CD47 in CMT 93 cells. ß2-Microglobulin was used as loading control. (C) Representative histograms of macrophage phagocytosis for the same populations of cells *in vivo*. (D) Phagocytic indices of CD47KD#1 and CD47KD#2 CMT93 cells (black bars) and scrambled CMT93 cells (white bar) from the *in vivo* phagocytosis assay (n = 7).

**SUPPLEMENTARY FIGURE S2.** Immunofluorescent staining of CD47 and SIRPα expression in macrophages and cancer cells. Hepa1-6(A) and CMT93 (B) showed CD47 expression of tumor cell surface, whereas SIRPα was not. CD47 expression was detected in scramble Hepa1-6 and scramble CMT93. CD47KD Hepa1-6 and CD47KD CMT93 did not express CD47 protein on cell surface. Pre-incubation with anti-CD47mAb decreased CD47 expression of Hepa1-6 andCMT93. (C) Macrophages expressed both SIRPα and CD47. Microscopic images were demonstrated at 40 x magnification with a fluorescent microscope.

**SUPPLEMENTARY FIGURE S3.** Immunohistochemical analysis of CD47 expression in cancer cells of *CPC-APC* mice (40× magnification).
